# Supplementary material for: Association between oral microbiome and seven types of cancers in East Asian population: a two-sample Mendelian randomization analysis
Source: Front Mol Biosci. 2023 Nov 21;10:1327893. doi: 10.3389/fmolb.2023.1327893 (PMC10702768; doi:10.3389/fmolb.2023.1327893)
Supplement: Supplementary file 1 [file DataSheet1.ZIP › Supplementary Materials/MR plots of saliva/saliva═╝/colorectal cancer/pheno.2448_to_colorectal cancer_forest.pdf]

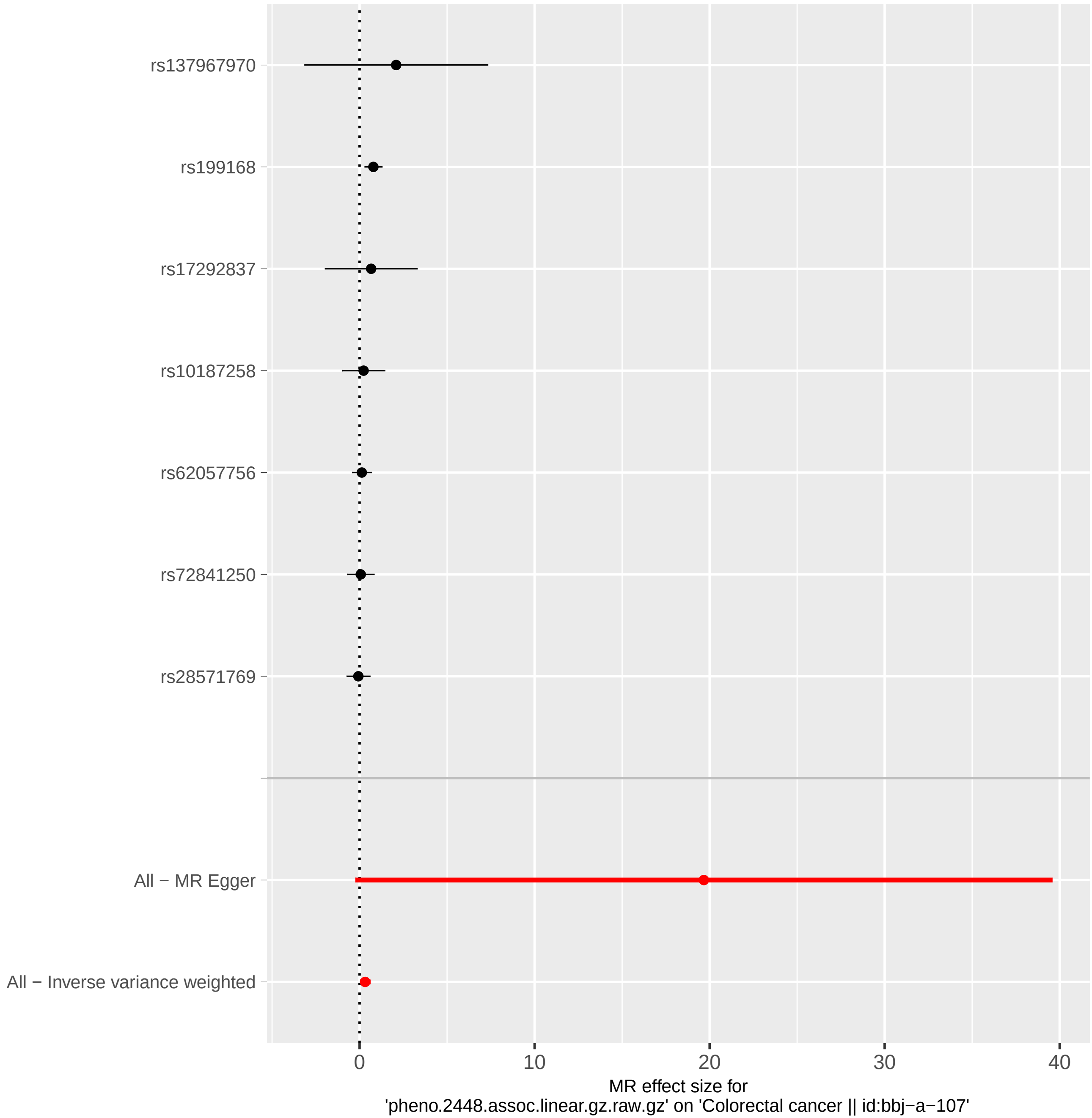
rs137967970
rs199168
rs17292837
rs10187258
rs62057756
rs72841250
rs28571769
All − MR Egger
All − Inverse variance weighted
0
10
20
30
40
MR effect size for
'pheno.2448.assoc.linear.gz.raw.gz' on 'Colorectal cancer || id:bbj−a−107'
